# Obstructive Jaundice Secondary to Extramedullary Hematopoiesis

**DOI:** 10.7759/cureus.17927

**Published:** 2021-09-13

**Authors:** Syed Kamran, Ammar Al-Obaidi, Yamama Al-Khazraji, Joel Alderson, Pavan S Reddy

**Affiliations:** 1 Internal Medicine, Kansas University School of Medicine, Wichita, USA; 2 Internal Medicine, Metropolitan Hospital Center, New York City, USA; 3 Pathology, Ascension Via Christi St. Francis Hospital, Wichita, USA

**Keywords:** obstructive jaundice, hematopoiesis, extramedullary hematopoiesis, hyperbilirubinemia, bone marrow biopsy, bilirubin, anemia

## Abstract

Extramedullary hematopoiesis (EMH) is the development of hematopoietic tissue outside of the bone marrow. In adults, the bone marrow is the main site of hematopoiesis. When this process occurs outside of the bone marrow, it is a sign of disease or deficiency. Clinically, the findings of EMH may be diverse. One rare complication that can arise from EMH is obstructive jaundice. This occurs when there is a blockage of bile flow leading to retention of bilirubin in hepatocytes. Identifying the markers of EMH and obstructive jaundice is important for optimizing positive outcomes. While often asymptomatic, EMH can be deadly if left untreated. In this case, we present a patient with obstructive jaundice secondary to EMH.

## Introduction

Extramedullary hematopoiesis (EMH) is the development of hematopoietic tissue outside of the bone marrow. This process is essential in early fetal life, however, is considered abnormal once it occurs after birth [1]. In adults, the bone marrow is the main site of hematopoiesis, and the continuous production of blood is maintained by pluripotent cells [2]. When this process occurs outside of the bone marrow, it is a sign of disease or deficiency. Pathologic sites of EMH generally include the liver and spleen, but may also include non-hepatosplenic sites such as the kidneys and adrenal glands, and less commonly the gastrointestinal tract, skin, pericardium, and brain [2,3]. This disease is often associated with other hematologic abnormalities such as myelofibrosis, chronic myeloid leukemia, or even myelodysplastic syndrome. Oftentimes, an associated disease is never identified [4]. Clinically, the findings of EMH may be diverse. Most may be found incidentally, such as pleural effusion, ascites, neurologic deficits, and acute renal failure [1]. Intestinal obstruction also commonly presents with ascites. A hematopoietic mass can cause symptoms resulting from the stricture of organs and compression of nearby structures [5].

Obstructive jaundice, a symptom of a wide variety of diseases, causes the accumulation of conjugated and unconjugated bilirubin in the serum. The blockage of bile flow leads to retention of the bilirubin in hepatocytes which can diffuse out into plasma [6]. The serum markers for bilirubin and alkaline phosphatase can be used to determine the significance of the hepatobiliary obstruction [7]. The differential diagnosis for obstructive jaundice is wide and age-dependent. While it is most commonly seen with cholelithiasis, after invasive procedures, or an intrahepatic cause, it can also sometimes occur due to an external tumor [8]. This case report explores the rare case of EMH causing obstructive jaundice.

## Case presentation

A 61-year-old male with a past medical history of type 2 non-insulin-dependent diabetes mellitus was admitted to the inpatient internal medicine service for nonspecific symptoms of generalized fatigue, weakness, poor appetite, and shortness of breath on exertion. The patient reports symptoms onset began two weeks prior to his initial presentation with fairly acute onset. Physical examination was remarkable only for jaundice, icteric sclera, and mild, generalized abdominal tenderness. Vital sign measurements were unremarkable. Initial laboratory data revealed pancytopenia and abnormal liver function test consistent with hyperbilirubinemia, elevated transaminases with a cholestatic pattern (Table 1).

**Table 1:** Lab results of the patient MCV - mean corpuscular volume, RDW - RBC distribution width; WBC - white blood cell

	Result	Normal Range
Total bilirubin	3.1 mg/dL	0.2-1.2 mg/dL
Alkaline phosphatase	160 µ/L	40-150 µ/L
Asparate aminotranferas (AST)	82 µ/L	5-34 µ/L
Alanine aminotransferase (ALT)	169 µ/L	0-55 µ/L
Lactate dehydrogenase (LDH)	2,409 U/L	125-220 µ/L
Blood urea nitrogen (BUN)	52 mg/dL	8-26 mg/dL
Creatinine	1.69 mg/dL	0.72-1.25 mg/dL
Hemoglobin	6.4 g/dL	14-18 g/dL
Hematocrit	18.40%	42%-52%
MCV	88.9 fl	80-100 fl
RDW	13.20%	11.5%-15.4%
Platelet count	97,000/µL	150,000-450,000/µL
WBC	2.4 x 10^3/µL	4.8-10.8 x 10^3/µL

**Figure 1:**
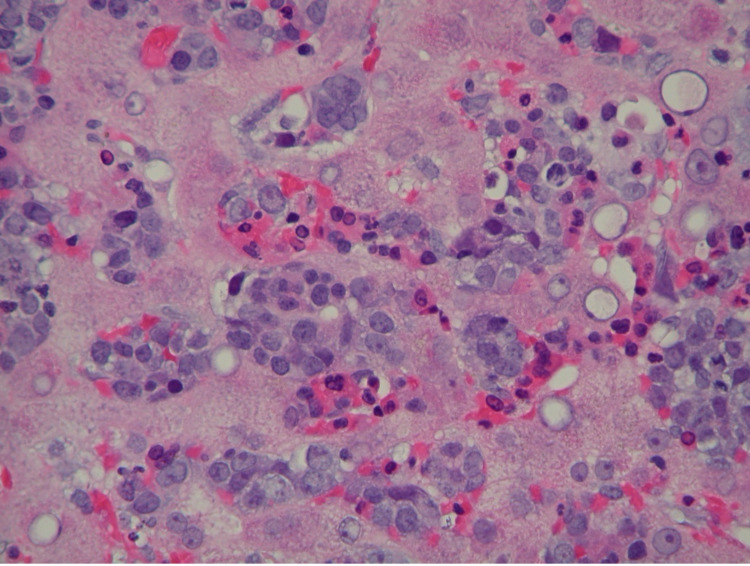
Liver hematoxylin and eosin stain showing marked involvement with extramedullary hematopoietic cells, mainly erythroid precursors, with hepatocanalicular cholestasis, periportal, and focal bridging fibrosis.

An automated differential showed 1% metamyelocytes, 66% neutrophils, 26% lymphocytes, 1%monocytes, 0% eosinophils, and 1% basophils. On peripheral blood smear, there was increased polychromasia, nucleated red blood cells, and erythroid precursors (Figure 1).

**Figure 2:**
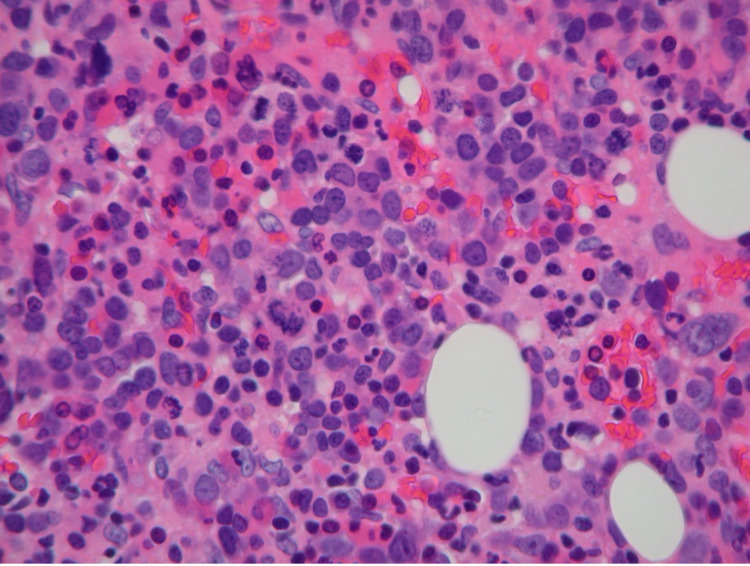
Bone marrow hematoxylin and eosin stain showing increased marrow cellularity with erythroid predominant trilineage hematopoiesis, dyserythropoiesis, and blast cells.

Because of the pancytopenia with circulating blasts, a bone marrow pathology was suspected. A manual differential on the peripheral blood smear showed 7.5% blasts, 1.5% myelocytes, 0.5% metamyelocytes, 64.8% neutrophils, 19.1% lymphocytes, 7% monocytes. Bone marrow biopsy was obtained and showed approximately 95% cellular marrow with erythroid predominant trilineage hematopoiesis and dyserythropoiesis, 11.6% blasts, no morphologic or immunophenotypic evidence of metastatic carcinoma, granulomas, or lymphoid aggregates. The bony trabeculae and blood vessels appeared unremarkable. Megakaryocytes were adequately present. An iron stain was performed on the bone marrow aspirate and core biopsy showed adequate storage of iron. No ringed sideroblasts were seen. Such changes were consistent with myelodysplastic syndrome with excess blasts type-2 (Figure 2). Flow cytometry was performed on peripheral blood and was consistent with a clonal myeloid neoplasm without immunophenotypic evidence of non-Hodgkin lymphoma.

The patient underwent aggressive fluid resuscitation, platelets, and blood transfusions to maintain hemodynamic stability. Decitabine was initially planned but was not started due to a persistent increase in liver markers, worsening jaundice, and the overall functional status of the patient. He rapidly deteriorated and exhibited signs of hepatic encephalopathy with liver function test of ALT 382 U/L, AST 120U/L, Alk Phos 190 U/L, INR 2.2, protein 4 g/dL, and bilirubin total 35.5 mg/dL with mostly direct bilirubin suggesting obstructive jaundice despite abdominal imaging revealed neither biliary obstruction nor dilation. However, magnetic resonance imaging revealed nodular changes of the liver consistent with cirrhosis associated with splenomegaly and sequela of portal hypertension. Laboratory data corresponded to a model for end-stage liver disease (MELD) score of 35. His iron studies were overall unremarkable. His Vitamin B12 and folate were also normal. Smooth muscle antibodies were normal. The serological marker for hepatitis B, hepatitis C, and human immunodeficiency virus (HIV) was negative.

**Figure 3:**
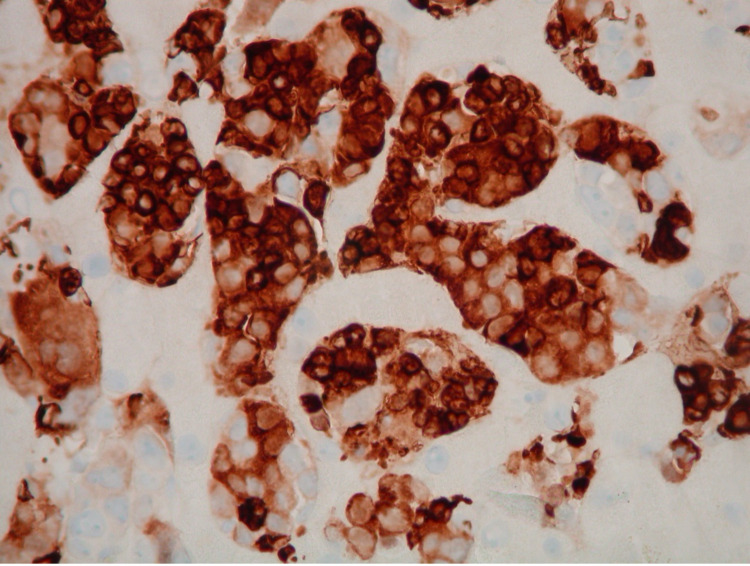
Hemoglobin A immunohistochemistry: the stain is highlighting erythroid cells.

Liver biopsy was subsequently obtained and found to be diffusely involved by marked EMH, predominantly erythroid, with hepatocanalicular cholestasis, patchy marked (4+) hemosiderosis in hepatocytes (parenchymal pattern), and periportal and focal bridging fibrosis (Figure 3). No infiltrating carcinoma was identified. Cytomegalovirus immunostain was negative.

Based on the above findings, a diagnosis of obstructive jaundice secondary to EMH was made. We could not arrive at a diagnosis for the EMH with the available evidence. The patient condition rapidly worsened and he was started on methylprednisonlone, piperacillin-tazobactam, allopurinol, lactulose, and rifaximin. Given the patient’s rapid decline in performance status and his decompensated liver failure disease, he was deemed not a good candidate for aggressive chemotherapy. The patient was discharged to hospice care 17 days after admission.

## Discussion

The early diagnosis of obstructive jaundice and EMH are paramount in optimizing positive outcomes. Evaluation of obstructive jaundice can be made with hepatic imaging such as an ultrasound, or a magnetic resonance cholangiopancreatography (MRCP) to search for signs of duct dilation [9]. If imaging is negative, further evaluation of hepatocellular injury and autoimmune markers can be further examined [2]. While fine-needle biopsy can lead to a definitive diagnosis of EMH, a presumptive diagnosis can be made with symptoms and imaging in some cases with bone involvement. [5]. Magnetic resonance imaging examination of long bones can show thinning of cortices, marrow expansion, and reconversion [2]. Interestingly, in this patient’s liver biopsy, besides the signs of EMH, he was also found to have prominent hepatocanalicular cholestasis, which likely resulted in his hyperbilirubinemia and obstructive jaundice [7]. 

Under stressful conditions, EMH can occur, and many various organs can become sites of hematopoiesis. While little is understood about the pathophysiology, it seems to occur most commonly due to tumors, anemia, infection, and metabolic stress [10]. It is not known if certain sites are linked to a distinct purpose. Since EMH may be asymptomatic in the early stages, identifying complications such as obstructive jaundice is key for survival. Goals for future studies include learning how hematopoietic stem cells can travel out of the bone marrow and begin functioning in extramedullary tissue. Understanding this concept could be beneficial for future stem cell transplantation treatment [11].

EMH presenting with obstructive jaundice is uncommon. Being aware of both the clinical spectrum of EMH, including obstructive jaundice, and the cytological findings are crucial for timely diagnoses [10]. If suspected, it is important to note a short differential of effects such as gastric outlet obstruction and acute appendicitis, both of which have been documented alongside EMH [1]. The treatment of choice for EMH is low-dose radiotherapy because hematopoietic tissues have extreme sensitivity to radiation [12]. It is also important to look for concurrent chronic anemia in the patient. Many EMH cases are thought to be a result of a compensatory process to combat a chronic, anemic state [1].

## Conclusions

While it is important to identify the markers of EMH and see the signs of obstructive jaundice, it is equally important to find the cause. EMH is known to cause organomegaly and tumor-like masses, which can lead to cord compression or respiratory failure. However, it is most often asymptomatic and microscopic. Further studies into its development for early detection would be beneficial to prevent poor outcomes. Identifying pertinent physical exam features and obtaining proper imaging to pair with lab findings of anemia or other deficiencies can be crucial steps in uncovering the disease early. It is unlikely there is one mechanism that sufficiently explains the causes of EMH, but rather it develops through multiple pathways at once.
